# Characterization of Mucosal Lesions in Crohn's Disease Scored With Capsule Endoscopy: A Systematic Review

**DOI:** 10.3389/fmed.2020.600095

**Published:** 2021-01-14

**Authors:** Miquel Marquès Camí, Alba Serracarbasa, Geert D'Haens, Mark Löwenberg

**Affiliations:** ^1^Servei d'Aparell Digestiu, Hospital Universitari Arnau de Vilanova, Lleida, Spain; ^2^Servei d'Aparell Digestiu, Hospital Universitari de Bellvitge, Hospitalet de Llobregat, Spain; ^3^Department of Gastroenterology and Hepatology, Amsterdam University Medical Centre, Location Academic Medical Centre, Amsterdam, Netherlands

**Keywords:** inflammatory bowel diseases, Crohn's disease, capsule endoscopy, intestine, small/diagnostic imaging, intestine, small/pathology

## Abstract

**Background and Aims:** There is little agreement on the nomenclature and description of Crohn's disease (CD) lesions that can be found in the small and large bowel using capsule endoscopy (CE). We performed a systematic review to identify mucosal lesions that have been described using CE in CD, in both the small bowel and colon, with the aim to make propositions to homogenize such descriptions.

**Methods:** A systematic literature search was conducted using Embase, Medline (OvidSP), and Cochrane Central on August 6, 2019. Clinical studies providing nomenclature and descriptions for small bowel and colonic inflammatory lesions using CE in CD were selected for data collection.

**Results:** In total, 851 articles were included for abstract screening out of which 219 were analyzed for full-text review. Twenty-two articles were selected for data extraction. Seven items, accompanied by clear descriptions, were found for the small bowel: i.e., ulcer, erosion, aphthoid lesion, edema, fissure, cobblestone appearance, and villous atrophy. No studies were found describing inflammatory items using CE in colonic CD.

**Conclusions:** The most frequently described CD lesions using CE were ulcers and erosions. Subjective interpretation of CE inflammatory findings plays an important role. Based on our findings, a range of suggestions regarding items and descriptions is made that might form the basis of a pan-enteric CE activity index.

## Introduction

Capsule endoscopy (CE) provides a reliable and non-invasive method to visualize the entire gastrointestinal tract. CE has a diagnostic yield of 50% to detect mucosal lesions in the small bowel of Crohn's disease (CD) patients (1). However, little agreement exists on how to describe such mucosal lesions using CE in CD. Capsule Endoscopy Structured Terminology (CEST) has been designed and published by international societies trying to seek consensus in interpreting and reporting small bowel CE examinations (2). Despite standardized terminology, descriptions vary considerably throughout different studies, and also interpretation of these findings varies widely among different observers.

Furthermore, criteria for small bowel CE-based activity assessment in CD vary considerably between different studies. The two available and validated endoscopic activity indices to assess small bowel CD activity [Capsule Endoscopy Crohn's Disease Activity Index (CECDAI) (3) and Lewis Score (LS) (4)] both rely on three parameters: inflammatory lesions, disease extension, and presence of strictures. In the LS, differentiation of ulcers from mucosal breaks, erosions and aphthoid lesions have been eliminated in order to develop a more user-friendly activity index. Moreover, this score provides an accurate description of inflammatory items (villous edema and ulcer). In contrast, the CECDAI does not provide item descriptions but grades findings from mild to severe and separates mucosal breaks into different sizes. Both activity scores contain inflammatory lesions with clinical significance according to a CE consensus meeting. However, only the LS provides clear descriptions for each item. Nevertheless, proposed descriptions were not consensually agreed upon.

On the other hand, validated scoring systems to assess colonic CD activity with CE are lacking. Hence, CE cannot be recommended yet to replace conventional colonoscopy in CD (5). In the past years, several studies showed that CE, using the second-generation colon capsule, is a safe and feasible tool to assess inflammatory activity in the small and large bowel (6–10). However, one study demonstrated an underestimation of the total ulcerated surface in the colon mainly because of insufficient bowel cleansing techniques compared to conventional endoscopy (6). Moreover, evidence suggests that CE is a useful technique to monitor post-operative CD patients (11). Nevertheless, all available CE studies used endoscopic activity indices, and no specific terminology for colonic findings has been proposed using CE.

In the treat-to-target era, where mucosal healing is an important treatment goal in CD, development of an activity index to assess the entire gastrointestinal tract, including the colon, is warranted. Recently, a pan-enteric index (CECDAIic) has been created demonstrating its usefulness in CD (12, 13). This particular CE activity score is based on the CECDAI and gives a comprehensive view of the entire intestinal tract but does not provide a clear description of inflammatory lesions.

Hence, validated scoring systems for pan-enteric CE that are accompanied by clear descriptions of items are lacking. As a first step, all CD-related mucosal lesions that have been reported in the literature should be characterized. Next, a consensus meeting consisting of CE experts should find agreement in the nomenclature and how to describe these items. As a final step, this might result in the development of a pan-enteric CE activity index. The development of such an index will likely decrease intra- and inter-observer disagreement.

Currently, scarce consensus exists on how to describe CD lesions detected by CE. In that regard, a Delphi consensus meeting took place consisting of small bowel CE experts. This working group proposed a set of items, together with clear descriptions, on how to describe small bowel CD lesions using CE (14), based on the LS and CECDAI.

With the aim of contributing to a uniform report of mucosal lesions, we performed a systematic review to identify all descriptions for mucosal inflammatory lesions using CE in the small and large bowels in CD patients, in order to make recommendations that might form the basis of a pan-enteric CE activity index.

## Methods

A systematic literature search was performed using the following databases on August 6, 2019: MEDLINE, EMBASE, and Cochrane library. The search strategy can be found as Supplementary Material. Two authors (MM and AS) independently screened all the articles by title and abstract, and when included by at least one of the authors for full-text revision the full article was analyzed. Any disagreements were resolved by discussion with a third author (ML) followed by a consensus meeting (ML, MM, and AS).

Study selection was carried out according to PICOS criteria for including and excluding studies. The inclusion criteria were as follows: description of CE items in the small bowel and/or the colon in patients with known or suspected CD who underwent CE for different indications [i.e., diagnosis (suspicion of CD) or staging, assessment of disease activity, or mucosal healing (in patients with known CD)]. Exclusion criteria were as follows: (1) provide CE items without description; (2) sample size <10 CD patients; (3) editorials, letters, review articles, meta-analyses, guidelines, meeting abstracts, and non-fully published data; (4) duplicated studies; and (5) other than English language. After the full-text selection, every CE item with its description was collected. Finally, we checked if the terminology was in line with international recommendations (2). We decided to exclude articles with <10 CD patients as a cut-off to assume the experience in CE in inflammatory bowel diseases. In that same line, we also checked if the study provided data regarding inter- and intra-observer agreement of each described item between CE observers.

## Results

**Figure 1:**
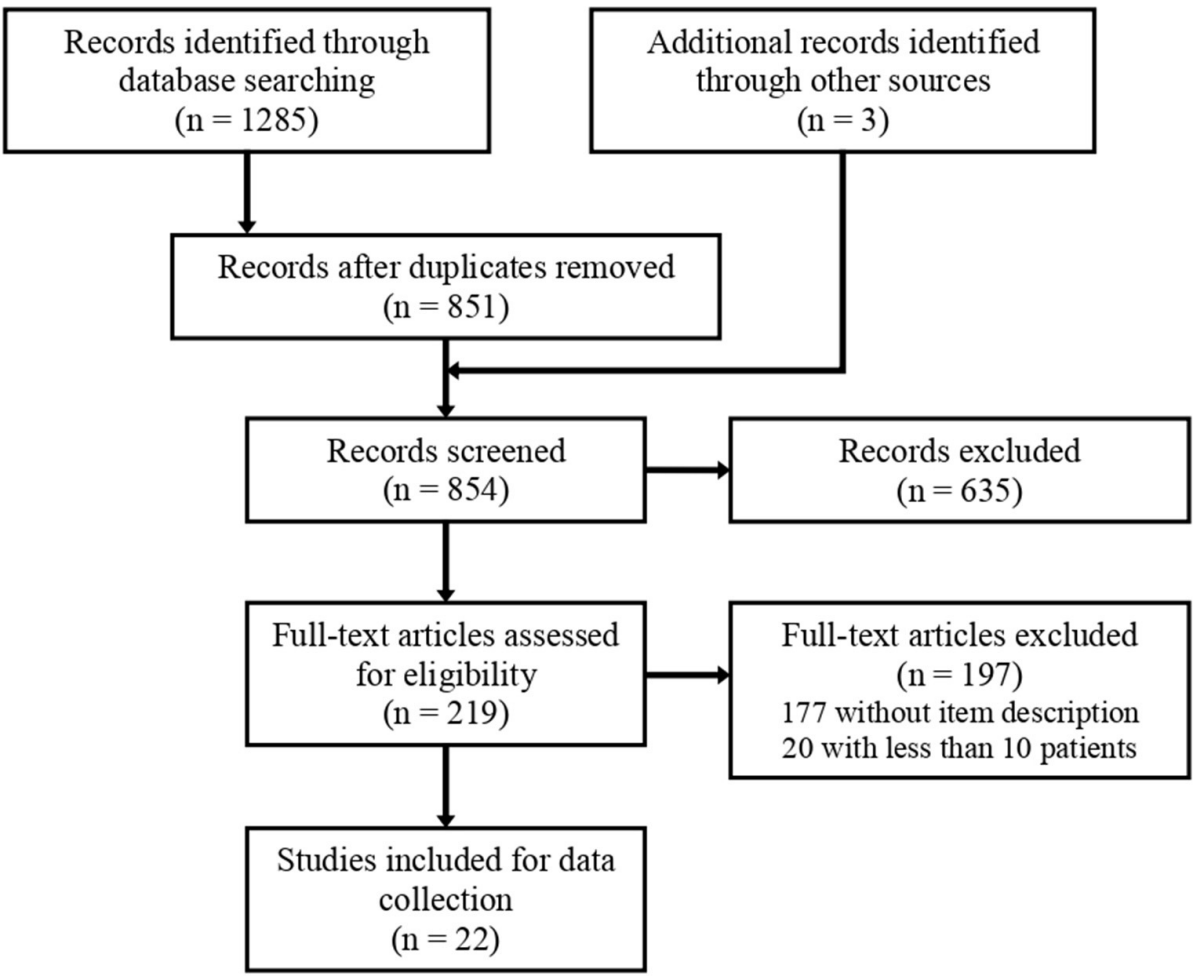
Flow diagram of the study selection process.

The literature search identified 1,285 records. Three additional records were identified through other sources. After removing duplicates, a total of 854 records were screened for inclusion. After screening titles and abstracts, 219 reports were selected for full-text review. After full-text review, 22 studies were included in the data collection process. A flowchart of the selection process is shown in Figure 1.

### Item Description Data

The selected studies provided an item description of the mucosal lesions with assumed clinical relevance in CD. All papers provided a priori specified item description before study performance. These definitions are shown in Table 1.

**Table 1:** Nomenclature used to specify inflammatory-related findings for CE in small bowel CD and the different descriptions given to each of them.

**Authors**	**Nomenclature and description**						
	**Ulcer**	**Erosion**	**Aphtha**	**Edema**	**Fissure**	**Cobblestone pattern**	**Villous atrophy**
Argüelles-Arias et al. (15)			Erythematous mucosa with a white central area				
Mow et al. (16)	White lesions within a crater and with surrounding erythema	Superficial white lesions with surrounding erythema					
Marmo et al. (17)	White lesions within a crater and with surrounding erythema	Superficial white lesions with surrounding erythema					
Efthymiou et al. (18)	*Aphtous ulcer*: white center and a red halo around it *Large ulcer:* All ulcers that are not apthous ulcers						
Gralnek et al. (4)	Mucosal breaks with white or yellow bases surrounded by red or pink collars.			Villous width is equal or greater than villous height			
Mehdizadeh et al. (19)	White lesions within a crater and with surrounding erythema	Superficial white lesions with surrounding erythema					
Figueireido et al. (20)	White lesions within a crater and with a surrounding erythema	Superficial white lesions with surrounding erythema					Circumscribed area of villous denudation
Jensen et al. (21)	Pale lesion within a crater representing a visible loss of mucosal substance.	Superficial and pale mucosal break surrounded by a red rim.	Superficial and pale mucosal break surrounded by a red rim.				
Mehdizadeh et al. (22)	White lesions within a crater and with surrounding erythema	Superficial white lesions with surrounding erythema					
Casciani et al. (23)	White lesions within a crater with surrounding erythema	Small superficial white lesions, even with surrounding erythema					
Di Nardo et al. (24)	White lesions within a crater with surrounding erythema	Small superficial white lesions, even with surrounding erythema					
Koulaouzidis et al. (25)	Any pale or yellow-based mucosal break surrounded by a red or a pink collar						
Esaki et al. (26)	Whitish crater surrounded by mucosal erythema presumably measuring over 5 mm	Superficial whitish lesion with surrounding erythema <5 mm in size					
Halling et al. (27)	Pale lesion within a crater representing a visible loss of mucosal substance		Superficial and pale mucosal break surrounded by a red rim		Longitudinal ulcers	Connected longitudinal and transversal fissures	
Höög et al. (28)	Mucosal breaks with white or yellow bases surrounded by red or pink collars.						
Kono et al. (29)		Small mucosal breaks of ≤ 3 mm	Small mucosal breaks of ≤ 3 mm				
Aloi et al. (30)	White lesions within a crater with surrounding erythema						
Urgesi et al. (31)						Multiple longitudinal ulcers running parallel and hill-like elevations due to submucosal swelling	
Hale et al. (32)	Mucosal breaks with white or yellow bases, surrounded by a pink or red collar						
Lee et al. (33)	White lesion within a crater, representing a visible loss of mucosal substance.	Superficial white lesions	A superficial pale mucosal break surrounded by a red rim				
Ninomiya et al. (34)	White lesions within a crater and with surrounding erythema	Superficial white lesions with surrounding erythema					
Esaki et al. (35)	Oval, longitudinal, circular or irregular mucosal defect >3 mm	Oval, longitudinal, circular or irregular mucosal break of <3 mm					

We found in total seven mucosal lesions with different descriptions. All items referred to small bowel CD lesions. The most frequently described items were ulcer (4, 16–28, 30, 32–35) and erosion (16, 17, 19–24, 26, 29, 33–35). Both definitions are characterized by a central mucosal defect with surrounding focal erythema and are distinguished from each other based on their size and depth of the defect. Different terminology has been used concerning each feature: crater, white lesion, mucosal break, pale lesion, white/yellow base, loss of mucosal substance, red/pink collar, or red rim. Esaki and colleagues (35) also distinguished ulcers and erosions according to shape (i.e., oval, circular, longitudinal, and irregular).

For aphthoid lesion, three different definitions were found (15, 21, 27, 29, 33). These are comparable to erosion descriptions, specifying its superficiality or small size. The validation study of the well-known LS provided the description for the item edema, characterized by equal or greater villous width when compared to villous height (4). To describe cobblestone pattern, two definitions have been used that take into account the presence and disposition of longitudinal ulcers (27, 31). One single definition has been provided for fissure (27) and villous atrophy (20). We did not identify any description regarding colonic lesions. With reference to the CEST standardized nomenclature, all terms are included in these international recommendations except for fissure and cobblestone pattern.

### Inter- and Intra-observer Agreement Assessment for Each Described Lesion

Two studies assessed agreement between CE observers regarding lesion identification and description (4, 35). These results are summarized in Table 2. Kappa statistics (*k*) were used to measure inter-rater reliability, with values ranging from 0 (absence of agreement) to 1 (perfect agreement). They interpreted values <0.20 as slight, 0.21–0.40 as fair, 0.41–0.60 as moderate, 0.61–0.80 as good, and >0.81 as excellent agreement.

**Table 2:** Studies assessing CE readers' agreement in relation to particular small bowel lesions and the grade of agreement obtained for each of them.

	**CE studies in CD patients**	**CE observers**	**Inter-observer agreement**	**Intra-observer agreement**
Gralnek et al. (4)	34	4	Ulcer: good Edema: moderate	NA
Esaki et al. (35)	25	4	Ulcer: Oval: slight to moderate Longitudinal: slight to moderate Irregular: fair to good Circular: fair to moderate Erosion: slight to moderate	Ulcer: Oval: fair Longitudinal: good Irregular: moderate Circular: good Erosion: moderate

Esaki and collaborators (35) distinguished ulcers and erosions according to four shapes. They evaluated the inter-observer agreement between one expert capsule endoscopist and three observers, analyzing small bowel CE results obtained from 25 CD patients. A slight to moderate agreement was found for oval and longitudinal ulcers, fair to good agreement for irregular ulcers, and fair to moderate agreement for circular ulcers. The authors detected slight to moderate agreement for overall erosion forms. The intra-observer agreement for the expert capsule endoscopist was fair, good, moderate, and good for oval, longitudinal, irregular, and circular ulcers, respectively. Intra-observer agreement was moderate for all erosion shapes.

Gralnek and colleagues (4) found moderate agreement for villous edema and good agreement for ulcer detection between four CE observers who analyzed 34 CE studies from CD patients.

Furthermore, four studies assessed observer agreement with regard to global small bowel inflammation by means of different small bowel CD scoring but not for any particular lesion (4, 17, 21, 31).

## Discussion

Currently, there is no consensus on how to describe small bowel and colonic lesions that can be found with CE in CD patients. We aimed to identify all mucosal lesions that have been described, both in the small bowel and in the colon, seeking common points in terms and descriptions to homogenize definition of mucosal lesions. Studies that lacked a clear description of mucosal lesions were excluded.

The most frequently described items related to inflammation in the small bowel of CD patients were ulcers and erosions. Both items are distinguished based on size or depth of the lesion, expressed by the presence of a crater or mucosal break. We propose to avoid this distinction based on size estimation, because objective measurement tools in CE are lacking and CE layout in relation to lesions when assessing a video may determine its interpretation. It should also be noted that the term *mucosal break* could lead to confusion, since it is generally used as a way to describe loss of mucosal substance, but some authors use it as a way to establish a deeper mucosal defect. We encourage to avoid this description of deep mucosal defect and propose to use other descriptors in that regard, such as *crater*. Moreover, the shape-based description approach by Esaki and coworkers could be counter-productive, because this is a subjective evaluation that might result in intra- and inter-observer disagreement.

With conventional endoscopy, an aphthoid lesion has been traditionally considered different from an erosion, since an aphthoid lesion has been seen as a flat or elevated lesion and an erosion as an excavated lesion covered by fibrin material. However, an aphthoid lesion is not included in the recommendations for endoscopy terminology of the World Endoscopy Organization (36). In the CEST, these two terms are classified separately, and the term aphthoid lesion is also considered an excavated lesion. Moreover, the CECDAI includes both terms with no attribute assessment. Therefore, we propose that these two terms could be used indistinctly.

The term edema is included in the CEST as a mucosal feature and is used in the two available validated activity indices, but only the LS validation study provides a description, testing its reproducibility with moderate inter-observer agreement but with no intra-observer agreement evaluation. Since the LS has been widely accepted for small bowel assessment in CD, few authors have insisted on changing this item description.

Fissure and cobblestone pattern are not included in the CEST. Halling et al. use the term fissure to describe longitudinal ulcers, and, due to its redundancy, we think it should be avoided. The cobblestone pattern definition provided by Urgesi and colleagues seems to be more accurate than the one provided by Halling and coworkers, since the Japanese criteria for CD diagnosis proposes the definition only by the presence of longitudinal ulcers when diagnosing CD (37), for both ileal and colonic diseases. Otherwise, it has always been related to a severe affectation, and it rarely appears in other inflammatory bowel diseases. As for colonoscopy, well-known activity indices (SES-CD and CDEIS) do not include cobblestone pattern as a diagnostic criteria by itself; hence, we think it should be avoided for inclusion in small bowel disease CE index.

We found one single definition of villous atrophy. The CEST uses the term *atrophy* when referring to mucosal aspect, not to villi, and applies the labels of shape and color concerning villi appearance. A Delphi consensus meeting (14) proposed to describe the absence of villi with the term *denudation*, with no reference to mucosal atrophy. Nevertheless, the group describing the LS eliminated *denuded mucosa*, because it was considered an item unable to be judged objectively and with perceived lack of clinical significance.

No studies were found that described colonic lesions with CE in CD. Most of these studies used endoscopic activity indices to score disease activity. The CECDAIic pan-enteric score validation study extrapolated to colon the inflammatory indicator used in the CECDAI for small bowel disease (12), with no colonic lesions description. Future studies are warranted using colon CE and pan-enteric CE in CD patients in order to better characterize mucosal lesions that can be found in the whole intestine. It will be of great importance to reach consensus between experts in characterizing mucosal lesions to improve agreement between readers using CE in CD patients. Additionally, these will help in better CE training, optimization of the learning curve, and broad implementation of CE in clinical practice.

Here, we mainly focus on inflammatory item description, working toward a more objective nomenclature and description. Of note, we did not analyze items that were related to stenosis, because its general definition implies a delay or withholding of CE rather than describing the mucosal pattern, even if it may be related with deep ulcers or edematous tissue. Likewise, we did not investigate studies focusing on the clinical relevance of mucosal lesions that can be detected by CE. Theoretically, the lesions to be considered suitable for scoring should contribute to clinical symptoms, correlate with endoscopic activity scores and with biological markers, and have a good rate of responsiveness on treatment outcomes. Beyond this point, the general line in the practice is to describe clinical relevance once different items have been included in a global score. The LS and the CECDAI both demonstrated its usefulness in diagnosing CD (38, 39) as well as staging (40) and monitoring CD patients (41, 42). Moreover, correlations between these scores and inflammatory biomarkers (fecal calprotectin and C-reactive protein) have been shown (43, 44). Likewise, the above-cited expert Delphi consensus about nomenclature and description of small bowel lesions only took into account small bowel items that were part of the LS and CECDAI. As stated by the authors, this consensus meeting did not assess the clinical relevance of such lesions. We are also aware that we may have left behind information regarding clinical relevance in studies not providing a clear description of mucosal lesions.

In conclusion, this robust systematic review identifies mucosal lesions that have been described using CE in CD in the small and large bowels. Personal interpretation plays an important role in describing these mucosal lesions. Here, we make suggestions to homogenize description of mucosal lesions detected by CE in CD. We suggest that to avoid ulcerative lesion distinction based on size or shape, the terms erosion and aphtoid lesion may be used indistinctly, the term *mucosal crater* should be avoided when describing an ulcer since it may be confusing, and the items fissure and cobblestone pattern might be of unnecessary redundancy and should be avoided. This manuscript may serve as a starting point to reach consensus between experts and might contribute to the development of a pan-enteric CE activity index.

## Data Availability Statement

The original contributions presented in the study are included in the article/Supplementary Material, further inquiries can be directed to the corresponding author.

## Author Contributions

MM, GD'H, and ML developed the study design. MM and AS carried out the search process and data extraction and wrote the draft. All co-authors have reviewed and corrected the draft. ML gave the final approval for the submission. All authors have contributed to and agreed on the content of the manuscript.

## Conflict of Interest

The authors declare that the research was conducted in the absence of any commercial or financial relationships that could be construed as a potential conflict of interest.
