# Supplementary material for: Characterization of Mucosal Lesions in Crohn's Disease Scored With Capsule Endoscopy: A Systematic Review
Source: Front Med (Lausanne). 2021 Jan 14;7:600095. doi: 10.3389/fmed.2020.600095 (PMC7840780; doi:10.3389/fmed.2020.600095)
Supplement: Supplementary file 1 [file Data_Sheet_1.docx]

**Literature search strategy on 6 august 2019**

**PubMed**

464 hits:

("Crohn Disease"[Mesh] OR crohn*[tiab])

AND

("Capsule Endoscopes"[Mesh] OR "Capsule Endoscopy"[Mesh] OR capsule endoscop*[tiab] OR  capsule enteroscop*[tiab] OR pillcam[tiab] OR video capsule*[tiab])

NOT

("Review" [Publication Type] OR "Systematic Review" [Publication Type] OR "Meta-Analysis" [Publication Type] OR "Case Reports" [Publication Type] OR "Editorial" [Publication Type] OR "Comment" [Publication Type] OR "Letter" [Publication Type] OR "Congress" [Publication Type] OR systematic review[ti] OR meta-analys*[ti] OR metaanalys*[ti] OR review[ti] OR case report[ti] OR letter[ti] OR editorial[ti])

**EMBASE (Ovid)**

Database(s): Embase Classic+Embase 1947 to 2019 August 05 

| # | Searches | Results |
| --- | --- | --- |
| 1 | Crohn disease/ or crohn*.ti,ab,kw. | 100412 |
| 2 | capsule endoscopy/ or capsule endoscope/ or (capsule endoscop* or capsule enteroscop* or pillcam or video capsule*).ti,ab,kw. | 9743 |
| 3 | 1 and 2 | 2341 |
| 4 | exp "review"/ or exp meta analysis/ or case report/ or editorial/ or letter/ or exp conference paper/ or (systematic review or meta-analys* or metaanalys* or review or case report or letter or editorial).ti. | 7490141 |
| 5 | 3 not 4 | 1491 |
| 6 | limit 5 to conference abstract status | 760 |
| 7 | 5 not 6 | 731 |

**Cochrane Central Register of Controlled Trials**

ID Search Hits

#1 (crohn*):ti,ab,kw 4566

#2 (capsule endoscop* or capsule enteroscop* or pillcam or video capsule*):ti,ab,kw 856

#3 #1 and #2 in Trials 90

**Summary**

| Databases: | Before deduplication | After deduplication |
| --- | --- | --- |
| PubMed, Embase (Ovid), Cochrane CENTRAL |  |  |
| Total | 1285 | 851 |
